# Health Risk Assessment of Trace Elements in Soil for People Living and Working in a Mining Area

**DOI:** 10.1155/2021/9976048

**Published:** 2021-07-02

**Authors:** Erasto Focus, Mwemezi J. Rwiza, Najat K. Mohammed, Firmi P. Banzi

**Affiliations:** ^1^The Nelson Mandela African Institution of Science and Technology (NM-AIST), P.O. Box 447, Arusha, Tanzania; ^2^Dar Es Salaam Institution of Technology (DIT), P.O. Box 2525, Dar Es Salaam, Tanzania; ^3^Tanzania Atomic Energy Commission (TAEC), P.O. Box 743, Arusha, Tanzania

## Abstract

The present study used soils collected from a small-scale gold mine area to determine the health risks due to trace elements to the at-risk population in the study area. The work involved 74 soil samples from four sampling categories: 29 samples were from the mining pits (MD), 18 samples from the first washing area (WA), 17 samples from the second washing area (WB), and 10 samples from the control area (C). All samples were analyzed for Cr, Cu, As, Pb, Cd, Co, Ni, Zn, and Hg using the Energy Dispersive X-Ray Florescence (ED-XRF) method. Trace element levels were found to vary across the four sampling categories. The concentrations of trace elements recorded from different sampling categories varied in an increasing order of MD > WA > WB > C. Mercury was detected in the highest levels (*max*. 3.72 ± 0.15) at WB while it was not detected in the samples from C. Samples from MD indicated that Cu (*max*. 737.66 ± 1.3 mg/kg) was found in the highest levels whereas Hg (*mean* = 0.007 mg/kg) was the lowest. At WA, Cu (max. = 178.97 ± 2.46 mg/kg) registered the highest average concentration while Hg (mean = 0.05 mg/kg) had the lowest concentration. For WB, Cu (max. = 230.66 ± 3.99 mg/kg) was found in the highest concentration. The hazard index value for all exposure routes was found to be 1.77, making noncarcinogenic effects significant to the adult population. For children, the hazard index value was 9.11, showing a severe noncarcinogenic effect on children living in the study area. For the noncancer effects through the inhalation pathway, the risk posed by Ni, Cu, Zn, and Pb was negligible for both adults and children, while Co posed the highest noncancer risk for children. Cobalt also indicated the highest noncancer risk for children through the dermal pathway, while As indicated the highest noncancer risk to children through ingestion. For the cancer risk, the adults were more at risk compared to children, except for As and Co through the dermal pathway posing the highest threat. Trace element concentrations, hazard quotient, and hazard index values indicated that the area was polluted and that noncarcinogenic and carcinogenic effects on residents and miners were significant. Therefore, there is a need to put in place mining regulations aimed at protecting the at-risk human population in the study area.

## 1. Introduction

The mining industry plays a significant role in the economic development of countries. The economic contribution is usually via the employment of skilled and nonskilled personnel and foreign income earnings that are necessary for national economic development [1]. However, mining operations are known to have harmful effects on both environmental and human health [2]. Mining operations are usually categorized in small-, medium-, and large-scale based on technology, labor, and capital investment necessities. To this end, emphasis in the mining sector should also be placed on the sustainability of the natural environment and human health management [3].

Mining operations have been associated with the elevation of trace elements that could have been at natural background levels before mining activities [4]. Essentially, all living systems require variable amounts of some elements to perform unique roles as sources of minerals and vitamins in the functioning of the human body but become toxic at higher levels [5, 6]. Some elements such as Pb, Cd, and As have no well-known beneficial function in the human body but are known to be toxic even at low concentrations [7, 8]. When absorbed by the body, trace elements accumulate in vital organs such as the liver, lungs, kidneys, brain, and bones for years, causing severe health problems [9]. Thus, the United States Agency for Toxic Substances and Disease Registry names As, Pb, and Hg as three major elements of concern to human and environmental health [8, 10].

Arsenic (in particular, As-III), for example, is considered a human cancer-causing agent at very low levels of exposure [11]. Prolonged exposure to As has also been linked to peripheral nerve mutilation that might cause diabetes [12]. Pb is considered a human danger and possibly a cancer-causing agent [13]. It also interrupts the normal functioning of the nervous and reproductive systems, joints, and kidneys and encourages renal tumors [8].

Cadmium is a well-known toxic element even at low levels that has also been considered as a likely cancer-causing agent [14]. Long-term exposure to Cd may also result in pulmonary effects including alveolitis, bronchiolitis, and emphysema [8, 9, 15]. Other health risks due to severe exposure to Cd include but are not limited to hypertension, kidney dysfunction, and bone fracture [16]. Furthermore, prolonged exposure to Cd has been linked to numerous detrimental health effects such as reduced fertility, arthritis, anemia, diabetes, cirrhosis, headaches, cardiovascular diseases, and stroke [17]. On the other hand, the severe ingestion of inorganic Hg can lead to hemorrhage, diarrhea, and gastrointestinal disorders [13]. Continuous and persistent exposure to Hg may well extremely affect the liver, kidney, and skin.

While chromium (Cr-III) is a vital constituent in the human body [13], chromium (Cr-VI) complexes are known to be cancer-causing agents. Inhalation of high levels of Cr-VI can also lead to shortness of breath and asthma, whereas long-term exposure to Cr-VI may cause damage to the kidney and liver. Ni, on the other hand, is well known to cause heart attacks, depression, kidney problems, hemorrhages, and cancer, both intestinal and oral [8, 18]. Although Cu and Zn are essential to human body function, they have been reported to cause noncarcinogenic effects to organs when taken in extremely excessive levels [19, 20]. Excessive use of Zn has been associated with weakening of reproduction and growth systems, while high Cu intake may cause liver damage [2].

Heavy metals and metalloids pose a critical risk not only to the adult human population but also to children in playgrounds, daycare centers, kindergartens, sport facilities, and schools and more so through the ingestion pathway [21]. Researchers in a recently published study applied gastrointestinal Unified Bio-accessibility Method (UBM) protocol to investigate the human health risk of As, Cd, Pb, Cr, Ni, Cu, and Zn associated with polluted dust and soils [21]. These researchers [*ibid*] found that polluted soils posed some noncancer and carcinogenic risk to children with soil pica behavior, i.e., geophagia problems. Compared to adults, children who live within active or abandoned mine sites are usually more at risk from heavy metals for both noncarcinogenic and carcinogenic pollution [22, 23]. It was found in a heavily mined site in Malaysia that exposure pathways to heavy metals from polluted soils ranked in the order ingestion > dermal > inhalation [22, 23].

It, therefore, follows that a pathway through which heavy metals can be transported within the environment and to organisms is important. There are several pathways of exposure including groundwater consumption, atmospheric deposition, and intake of polluted surface water. Routes of exposure of heavy metals into the human body include inhalation, ingestion, and body contact. The ingestion route is reported to be dominant for human exposure especially in the mining environments and children are reported to be at a higher risk than adults [22–25]. Furthermore, Veronica et al. [25, 26] reported health risk associated with the ingestion of considerable concentrations of trace elements especially to women and children when nonfood substances are ingested.

During the present study, different socioeconomic activities that could pose a risk to human health were ongoing; these included but were not limited to (1) the miners' families residing in the study area, (2) small guest housing businesses used by visitors for accommodation, and (3) smallholder farming, e.g., gardening, crop cultivation, animal keeping, and poultry production for resident families. Residents informed the researchers that, in the study area, it was normal for children to play with soils, whereas expecting mothers and mothers with infants were involved in washing the mined materials howbeit with poor protection. It was also noted that artisanal miners in the study area worked long hours without proper protective gear. Although the current situation is alarming, research information about trace element exposure and related health risks to miners and villagers is highly missing, not only in Tanzania but across the sub-Saharan Africa region. Therefore, the objective of the present study was to investigate the levels of different trace elements in soils collected from an exemplary small-scale mine and assess the health risks to the residents and mine workers.

## 2. Materials and Methods

### 2.1. Study Area

**Figure 1:**
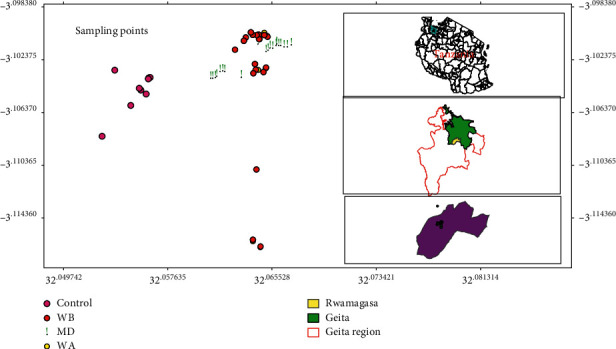
A map of Tanzania (top right) showing the Geita district (middle right), the study area (bottom right), and sampling points (left).

The present study was conducted at Rwamagasa mine in the Geita region of Tanzania. The Geita region lies on the southwest bank of Lake Victoria. Geologically, Geita is found in the gold-rich region, the Lake Victoria Goldfields (LVGF). Many of Tanzania's large-scale gold mining (LSGM) operations as well as artisanal and small-scale gold mining (ASGM) activities occur in these goldfields [27]. Thus, the study area was purposely chosen because of its long history of mineral extraction via both ASGM and LSGM in the gold-rich countries of East Africa. ASGM in the study area is done in different subdistricts including Nyarugusu, Rwamagasa, Nyakagwe, Nyamtondo, Iparamasa, Nyamalimbe, Kamena, and Mgusu [3, 28]. The present study was carried out in the Rwamagasa (Figure 1) subdistrict located at 3.1166°S and 32.0417°E with about 4000 ASGM miners [3]. The study sites may significantly provide desirable data related to trace element levels and distribution to gauge the impacts that the long existence of LSGM and ASGM has had on the environmental and human health of the area.

### 2.2. Sample Collection

A total of 74 soil samples were collected from the study area. Sampling points were grouped into four categories: WA (washing area A) referring to 18 soil samples taken from washing area A, WB (washing area B) referring to 17 soil samples taken from washing area B, MD referring to 29 soil samples taken from the mining pits, and C referring to 10 soil samples taken from a control site. At each sampling site, a sample was taken from three different points to represent the whole area.

All soil samples were placed in labeled polythene bags and transported to the Nelson Mandela African Institution of Science and Technology (NM-AIST) for preparation and storage and later were transferred to the Tanzania Atomic Energy Commission (TAEC) laboratory for analysis. For future referencing purposes, all sampling points were georeferenced using a handheld GPS receiver.

### 2.3. Sample Preparation

Soil samples were dried in an oven at a temperature of 50°C for 24 hours to remove moisture and obtain constant weight [29]. The soil samples were then crushed into a fine powder with a thoroughly cleaned mortar and pestle to obtain acceptable particle sizes passing through a 2 mm stainless steel sieve. Thereafter, 4 g of a sieved soil sample and 0.9 g of the starch binder were measured and mixed. A mixture of binder and sample was homogenized using a pulverizer for ten minutes before pressing into 32 mm diameter tablets with a die pellet maker.

### 2.4. Laboratory Measurements and Analysis

Soil samples were analyzed for trace elements using the Energy Dispersive X-Ray Florescence (ED-XRF (XOPOS, 4R0138, Kleve-Germany)) method. Before ED-XRF analysis was performed, the software was calibrated. The pellet samples were put into the ED-XRF, where the elemental compositions and concentrations were measured. The ED-XRF method has the advantage of high sensitivity, nondestructive, and specificity for the correct detection and quantification of trace elements [8]. The excellence of the analytical data was assured by employing typical quality assurance procedures [8]. Each sample was analyzed in triplicate. After each category sample, a certified soil standard (Montana Soil 2711A) was run to check for contamination [8]. Trace element concentrations from the ED-XRF analysis were obtained in mg/kg and %; those obtained in percentages were converted into mg/kg as well.

Health risk assessments were performed using different mathematical models. The risk associated with ingestion of trace elements through soil (ADI_Ingestion_) was estimated using equation (1). While the health risk associated with inhalation of trace elements through soil (ADI_Inhalation_) particulates was estimated using equation (2). Equation (3) was used to evaluate the risks due to dermal contact with the soil (ADI_Derm_). The carcinogenic and noncarcinogenic risks were assessed using equations (4) and (5) for noncarcinogenic effects and equation (6) for carcinogenic effects, respectively [10, 30]:

(1) $$\begin{array}{c} {\text{ADI}}_{\text{Ingestion}}=C\times \text{RI}\times f\times \text{ED}\times \frac{F}{B}\times T, \end{array}$$

(2) $$\begin{array}{c} {\text{ADI}}_{\text{Inhalation}}=C\times \text{IR}\times f\times \frac{\text{ED}}{b}\times T\times \text{PEf}, \end{array}$$

(3) $$\begin{array}{c} {\text{ADI}}_{\text{Derm}}=C\times \text{ESA}\times \text{FES}\times \text{SAF}\times \text{ABS}\times f\times \text{ED}\times \frac{F}{B}\times T, \end{array}$$

(4) $$\begin{array}{c} \text{HQ}=\frac{{\text{ADI}}_{\text{Route}}}{\text{RD}}, \end{array}$$

(5) $$\begin{array}{c} \text{HI}=\sum_{i=1}^{n}{\text{HQ}}_{i}=\sum_{i=1}^{n}\frac{{\text{ADI}}_{i}}{{\text{RD}}_{i}}, \end{array}$$

(6) $$\begin{array}{c} {\text{Risk}}_{\text{pathway}}=\sum_{i=1}^{n}{\text{ADI}}_{i}{\text{CSF}}_{i}, \end{array}$$

where *C* is the concentration of trace element in soil, RI is the ingestion rate, *f* is the exposure frequency, *F* is the conversion factor, *B* is the body weight, *T* is the period over which the dose is averaged, ED is the exposure duration, PEf is the particulate emission factor, ESA is the exposure skin area, FES is the fraction of dermal exposure ratio to soil, SAF is the soil adherence factor, ABS is the fraction of the applied dose absorbed across the skin, RD is the reference dose of a specific chemical, and CSF is the cancer slope factor. The exposure parameters used in this study are presented in Table 1 [32].

Additionally, the cancer slope factor and the reference doses for different trace elements are presented in Table 2 [2].

### 2.5. Quality Control

To assess the accuracy provided by the ED-XRF technique, the Montana soil 2711A Standard Reference Material (SRM) obtained from the National Institute of Standards and Technology (NIST) was also prepared and analyzed under comparable experimental conditions as unknown samples. The levels obtained in the standard reference soil for every trace element were related to the certified values of the similar trace element in a sample to form the level of agreement between the certified and measured values. For the trace elements studied, the deviation between the certified and measured concentrations lied within ± 9%.

Moreover, the starch binder was used to give the homogeneity of the sample. The binder was used throughout the analysis to avoid any interference and/or significant contamination in the analysis process, which could result in misinterpretation of trace element concentrations. The analytical outcomes of the binder material showed that there was no significant contamination or interference for every analyte.

### 2.6. Limitations of the Study

A study related to exposure and human health risk would greatly benefit from samples collected from human subjects. Risk assessment studies that use blood, nails, hair, and other human samples would reach better conclusions than those that rely on only environmental samples. Another limitation of the present study is that concentrations of elements in soils were determined and used as the basis for risk analysis. However, having high concentrations of elements in soils does not always mean that those elements are bioavailable for absorption. The present study recommends further studies in the area that would look into soil extraction of the bioavailable fractions of the studied metals.

## 3. Results

### 3.1. Soil Trace Elements

The average levels of trace elements (mg/kg) from the different sampling categories are presented in Table 3.

The concentrations of trace elements from different sampling categories were recorded with varying levels (Supplementary Material Tables S1to S4). The average levels of trace elements in the mining pits varied in an increasing order from Cu with the highest level ranging from 51.86 ± 2.86 to 737.66 ± 1.30 mg/kg and an average value of 177.16 mg/kg to Hg with the lowest levels between 0 and 3.72 mg/kg equivalent to an average value of 0.07 mg/kg. The trend of the intermediate elements showed a clear Zn > Ni > Cr > Co > Cd > Pb > As > Hg, increasing elemental concentrations with levels ranging from 44.65 ± 4.77 to 131.61 ± 2.75 mg/kg averaging to 136.76 mg/kg for Zn; 44.65 ± 4.77 to 131.61 ± 2.75 mg/kg averaging to 96.82 mg/kg for Ni; 34.45 ± 2.09 to 113.23 ± 4.34 mg/kg averaging to 54.15 mg/kg for Cr; 10.63 ± 2.27 to 26.92 ± 0.52 mg/kg averaging to 17.75 mg/kg for Co; 0 to 6.36 ± 2.07 mg/kg averaging to 7.86 mg/kg for Cd; 1.60 ± 0.34 to 18.32 ± 1.53 mg/kg averaging to 6.03 mg/kg for Pb; and 0 to 36.11 ± 0.47 mg/kg averaging to 12.74 mg/kg for As, respectively.

On the other hand, the levels of trace elements in the first washing area (WA) varied in an increasing order of concentration levels with the pattern of Cu > Zn > Ni > Cr > Co > As > Pb > Cd > Hg (Table S2 in the Supplementary Material). The concentration levels recorded for each element include Cu (74.17 ± 2.58 to 178.97 ± 2.46 mg/kg) averaged 140.99 mg/kg Zn with an average of 115.46 mg/kg ranging from 39.78 ± 0.74 to 145.75 ± 6.84 mg/kg; Ni (46.49 ± 1.50 to 101.68 ± 3.31 mg/kg) with the average value of 83.59 mg/kg; Cr varied from 49.39 ± 4.91 to 120.19 ± 8.98 mg/kg with an average value of 77.59 mg/kg; Co (10.09 ± 0.90 to 38.73 ± 12.19 mg/kg) averaged 21.28 mg/kg; As (6.49 ± 2.20 to 24.09 ± 1.35 mg/kg) with an average value of 12.44 mg/kg; Pb (2.45 ± 0.20 to 17.54 ± 1.31 mg/kg) with the average value of 9.95 mg/kg; Cd levels ranging from 0 to 8.77 ± 1.05 mg/kg having the average value of 6.34 mg/kg; and Hg with the average of 0.05 mg/kg ranging from 0 to 0.053 mg/kg, respectively.

Furthermore, the levels of trace elements in the second washing area (WB) varied and increased in the order Cu > Zn > Cr > Ni > Co > As > Pb > Cd > Hg with the average range of 137.38 mg/kg for Cu ranging from 74.71 ± 3.86 to 230.66 ± 3.99 mg/kg; Zn (66.91 ± 2.68 to 157.85 ± 3.63 mg/kg) with the average of 98.78 mg/kg; Cr varied from 45.73 ± 2.86 to 280 ± 12.45 mg/kg having the average of 94.22 mg/kg; Ni (51.74 ± 1.71 to 105.59 ± 0.79 mg/kg) averaged to 72.19 mg/kg; Co (9.2 ± 0.76 to 36.76 ± 5.72 mg/kg) with the average value of 20.68 mg/kg; As with average value of 20.31 mg/kg with levels between 8.17 ± 1.29 and 60.54 ± 1.16 mg/kg; Pb (2.78 ± 0.48 to 34.19 ± 2.52 mg/kg) with the average 12.82 mg/kg; Cd varied from 0 to 8.48 ± 1.63 mg/kg with an average of 6.98 mg/kg; and Hg with an average concentration of 3.72 mg/kg, respectively (Table S3).

In comparison, the control area showed lower concentrations compared to MD, WA, and WB samples (Table S4). The control area concentration levels were also in an increasing pattern, in the order Cu > Ni > Zn > Cr > Co > Pb > Cd > As > Hg. The concentrations measured from the control area samples include Cu (60.84 ± 1.72 to 141.16 ± 3.84 mg/kg); Ni (50.81 ± 2.52 to 123.15 ± 0.33 mg/kg); Zn (34.48 ± 1.74 to 160.75 ± 3.19 mg/kg); Cr (40.09 ± 2.26 to 56.576 ± 4.62 mg/kg); Co (8.87 ± 1.09 to 30.21 ± 2.63 mg/kg); Pb (11.45 ± 0.74 to 16.46 ± 0.65 mg/kg); Cd (6.86 ± 1.84 to 9.27 ± 1.13 mg/kg); and As (0 to 5.14 ± 0.56 mg/kg). The average values for each element in the control area samples are presented in Table 3. Note that Hg was not detected in the control area soil samples that were investigated.

### 3.2. Noncarcinogenic Risk Assessment

**Figure 2:**
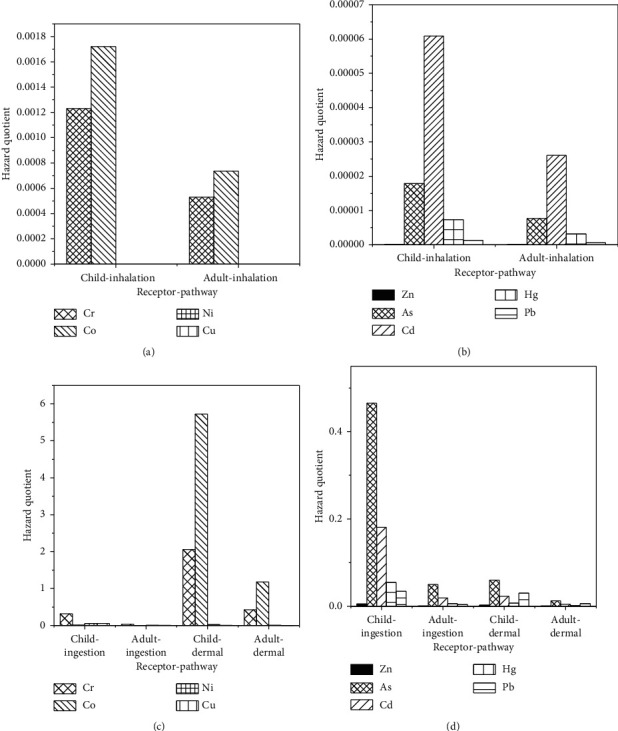
Hazard quotient values of selected trace elements for children and adults through the inhalation pathways (a, b) and via the ingestion and dermal pathways (c, d) in soil collected from the study area.

The noncarcinogenic risk for residents in the study area was evaluated based on children and adults based on the stipulated reference dose (R*f*D) values shown in Table 2 and the average daily intake (ADI) values presented in Table 4. The calculated data for the inhalation, ingestion, and dermal pathways are all presented in Figures 2(a)–2(d) in terms of hazard quotients (HQs).

### 3.3. Carcinogenic Risk Assessment

**Figure 3:**
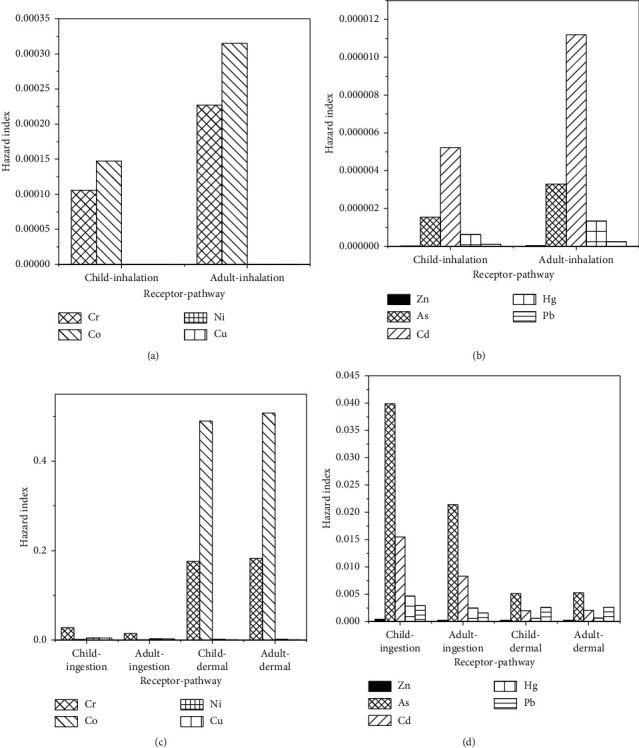
Cancer hazard indices of selected trace elements for children and adults through inhalation pathways (a, b) and through ingestion and dermal pathways (c, d) in soil from the study area.

The average dose intake in estimating the excess lifetime cancer risks for children and adults is presented in Table 5. The lifetime cancer risk analysis results are presented in Figures 3(a)–3(d).

## 4. Discussion

The levels of Co and Cd did not vary significantly in all four sampling categories (Table 3). This shows that the concentrations of Co and Cd in the analyzed samples were possibly from a geological source rather than being caused by anthropogenic activities. The results further showed that the lowest level of Cu (51.86 ± 2.86 mg/kg) was recorded at MD13 and a maximum level of 737.66 ± 1.30 mg/kg at MD8. Arsenic was not detected at MD8 while the maximum level of As of 36.11 ± 1.29 mg/kg was recorded at MD26. On the other hand, Ni recorded minimum and maximum levels of 44.65 ± 4.77 mg/kg and 131.61 ± 2.75 mg/kg at MD13 and MD29, respectively. Both Cu and Ni recorded minimum values of approximately equal magnitude at MD13. This phenomenon may indicate that at MD13, Cu and Ni were geologically found in trace amounts. Mercury was not detected in the control samples but was found to have values of 3.72 ± 0.15 mg/kg in samples from washing area B category, 0.05 ± 0.11 mg/kg for the washing area A, and 0.07 ± 0.15 mg/kg in the mining pits. The high value of mercury in washing area B suggests that miners may be using mercury for gold recovery. These high mercury levels in the washing area were in good agreement with an earlier study that showed high levels of mercury in biomonitored blood, urine, and hair from residents at Rwamagasa [14]. The recorded levels of trace elements in the mining area differ significantly from levels recorded in the control area (Table 3) suggesting that anthropogenic activities, e.g., mining, do influence the concentration and distribution of trace elements in different microenvironments. Arfaenia et al. [20] found that the levels of heavy metals were significantly higher in samples from industrial areas as compared to samples from urban environments, which is in line with the present study.

The values (bars) of the hazard quotients and cancer risks for some trace elements did not show up on the plots (Figures 2 and 3). This was because some elements such as As, Cd, Cu, Hg, Pb, and Zn were detected in small values compared to Co, Cr, and Ni. However, frequent and long-term exposure to even small amounts of carcinogenic elements such as As, Cd, Hg, and Pb may still cause serious human health problems [33].

The average daily intake (ADI) values in soil for noncarcinogenic effects (Table 4) indicated more effects on children than adults. For the three exposure routes considered, the total ADI was much greater for children than adults for the nine trace elements investigated. This indicated that children were more prone to noncarcinogenic risks than adults. These higher levels of ADI in children might be due to their living behaviors. In a different study, contaminated soils were found to pose more risk to children than they did to the adult members in a small-scale mining community [8]. The authors (*ibid*) also indicated that for both children and adults the ingestion pathway contributed highly to the noncancer risks followed by the dermal contact pathway. A similar trend was followed when carcinogenic effects were considered, with children at a higher risk than adults and ingestion being the dominant pathway (Table 5).

Generally, a large body of literature indicates that when the hazard quotient (HQ) and hazard index (HI) values are less than 1, there is no evident risk to the residents, but if these values exceed unity, there may be concerns for possible noncarcinogenic effects [2]. The total calculated HQ values for all elements for adults were less than one in the inhalation and ingestion routes, whereas the value of 1.64 was found for the dermal pathway. The observed high value through the dermal pathway may be indicative of a noncancer risk to the miners, soil washers, and residents at Rwamagasa. The hazard index for all pathways was equal to 1.77. This suggested that the residents were at the threat of noncarcinogenic effects. For children, the dermal and ingestion paths had HI and HQ values greater than 1, mostly driven by Cr and Co and gave a total HI of 9.11 for all three routes [8]. This high value indicated trace element pollution that may pose an appreciable noncancer health risk to children living around the study area [8]. These results were in good agreement with previous findings [8, 25]. The results also indicated that, for both adults and children, the dermal pathway adds the greatest to the noncarcinogenic risk, followed by the ingestion pathway, while inhalation was the least contributor to the risk as shown in Figures 2(a) and 2(b).

The cancer possibility was calculated based on the nine trace elements. As shown in Figure 3, As and Cr were found to be the most contributors to the cancer risk. The United States Environmental Protection Agency (USEPA) considers tolerable value for monitoring purposes a cancer risk of 1 × 10^−6^ [2, 8]. However, Tanzania has not yet developed the acceptable range for cancer risk regulatory purposes. In the present study, the carcinogenic risks for the adult and child population were found to be 3.42 × 10^−5^ and 6.16 × 10^−5^, respectively, which are higher than the tolerable limit. The results obtained from the present study show that children were consequently more at risk than adults. With the trace elements considered, the results indicated that soils collected from washing area B had higher levels of trace elements compared to the soils from other sampling categories. Studies done elsewhere indicated differentiated levels of elements for samples collected from areas with industrial activities when compared to concentrations in samples collected from residential and natural field areas [34].

Based on the suggested maximum permissible limits for Tanzania and other countries (Table 6), Cu, Co, Zn, and Cr were found to have the highest concentrations in the soils from the study area. Pb, Cd, and Hg had lower values less than the maximum allowable limits. However, the average value of 3.72 mg/kg for Hg in washing area B was much higher than the recommended limits by organizations and countries except for the United Kingdom which recommends a limit of 10 mg/kg.

## 5. Conclusions

The present study presents the health risks due to trace elements to people working and living in Rwamagasa, a small-scale gold mine area in Tanzania. With the trace elements involved, the results indicated that Cu had high concentrations in all sampling categories, followed by Zn. Cr and Co recorded high levels after Cu and Zn. Furthermore, Hg and As were not detected in the control area. However, in samples from washing area B, Hg had a higher value of 3.72 mg/kg than that measured in the mining pits and 0.05 mg/kg that was measured at washing area A. On the other hand, Pb recorded a high value of 14.02 mg/kg in control samples, higher than 6.03 mg/kg found in the mining pits. Based on the maximum acceptable limits recommended by Tanzania and other international organizations, Cu and Cd were found in appreciably high concentrations. For As, Cd, and Hg (in washing area B), their values in soil were much greater than the Tanzania maximum allowable limits. The results in the present study further indicated that, in both children and adults, the dermal route was the highest contributor to the noncarcinogenic risks, followed by the ingestion pathway. For the carcinogenic risk evaluation, the ingestion and dermal pathways contributed more. Thus, soils surrounding the study area were contaminated by trace elements, with high As, Cr, Cd, and Hg at washing area B. There is a need to put in place mining rules to protect miners and residents in Rwamagasa and other similar small mining communities.

## Figures and Tables

**Table 1:** Exposure parameters for children and adult population used in the present study [31].

Parameter	Unit	Adult	Child
Ingestion rate	mg/day	100	200
Exposure duration (ED)	Years	30	6
Body weight	kg	70	15
Inhalation rate	m^3^/day	20	10
Exposure frequency	Days/year	350	350
Dermal exposure ratio	—	0.61	0.61
Conversion factor	kg/mg	10^–6^	10^–6^
Dermal absorption factor	—	0.1	0.1
Skin surface area	cm^2^	5800	2100
Soil adherence factor	mg/cm^2^	0.07	0.2
*Average time*			
For carcinogenic	Days	365 × 70	365 × 70
For noncarcinogenic		365 × ED	365 × ED

**Table 2:** Reference doses (mg/kg day) and cancer slope factor for different pathways used in the present study [2].

Trace element	Oral RD	Dermal RD	Inhalation RD	Oral CSF	Dermal CSF	Inhalation CSF
	x 10^−4^		
Cr	30		0.3	0.5		4.1
Cu	370	240				
As	3	3	3	1.5	1.5	15
Hg	3	3	0.86			
Pb	36			0.0085		0.042
Cd	5	5	0.57			6.30
Zn	3000	750				
Ni	200	56				
Co	200	0.057	0.057			9.80

**Table 3:** Levels (mg/kg) of trace elements in soil from different categories in the present study.

Sample category	*n*	Average concentration (mg/kg) of trace elements in soils from different locations								
		Cr	Co	Ni	Cu	Zn	As	Cd	Hg	Pb
MD	29	54.15	17.75	96.82	177.16	136.76	12.74	7.86	0.07	6.03
WA	18	77.59	21.28	83.59	140.99	115.46	12.44	6.34	0.05	9.95
WB	17	94.22	20.68	72.19	137.38	98.78	20.31	6.98	3.72	12.82
C	10	49.79	18.85	84.86	104.36	80.62	0	7.47	0	13.7

**Table 4:** Average daily intake (ADI) values in soil from the study area for noncancer risk.

Receptor	Pathway	Average daily intake for trace elements (x 10^−6^ mg/kg/day)								
		Cr	Co	Ni	Cu	Zn	As	Cd	Hg	Pb
Child	Inhalation	0.037	0.009	0.041	0.075	0.058	0.005	0.003	0.001	0.005
	Ingestion	963	254	1080	1940	1500	140	90.3	16.4	123
	Dermal	123	32.6	138	249	192	17.9	11.6	2.1	15.7
	Total	1090	287	1210	2190	1690	157	102	18.5	138

Adult	Inhalation	0.0159	0.004	0.0177	0.032	0.0247	0.002	0.001	0.0003	0.002
	Ingestion	103	27.3	115	208	160	15	9.67	1.75	13.2
	Dermal	25.6	6.75	28.6	51.5	39.7	3.7	2.4	0.434	3.26
	Total	129	34	144	260	200	18.7	12.1	2.19	16.4

**Table 5:** Average daily intake (ADI) of soil samples from the study area for carcinogenic risk.

Receptor	Pathway	Average daily intake for trace elements (x 10^−6^ mg/kg/day)								
		Cr	Co	Ni	Cu	Zn	As	Cd	Hg	Pb
Child	Inhalation	0.003	0.001	0.004	0.01	0.01	0.001	0.0003	0.0001	0.0004
	Ingestion	82.5	21.8	92.3	166	128	12	7.74	1.4	10.5
	Dermal	10.6	2.79	11.8	21.3	16.4	1.53	0.991	0.18	1.35
	Total	93.1	24.6	104	188	145	13.5	8.73	1.58	11.9

Adult	Inhalation	0.007	0.002	0.008	0.02	0.01	0.001	0.001	0.0001	0.001
	Ingestion	44.2	11.7	49.4	89.1	68.7	6.41	4.14	0.751	5.64
	Dermal	11	2.89	12.2	22.1	1.59	1.59	1.03	0.186	1.4
	Total	55.2	14.6	61.7	111	8	8	5.17	0.938	7.03

**Table 6:** Allowable limits for trace element concentrations in soil (mg/kg) for different countries/organizations [8].

Country/organization	Maximum allowable limit^a^										
	Cr	Mn	Fe	Co	Ni	Cu	Zn	As	Cd	Hg	Pb
Tanzania	100	1.5	NI	NI	100	200	150	1	1	2	200
US EPA	11	NI	NI	270	72	NI	1100	NI	0.43	NI	200
FAO/WHO	100	NI	NI	50	50	100	300	20	3	NI	100
EU	75	140	300	NI	3	100	1	NI	3	NI	300
China	200	NI	NI	NI	50	100	250	30	0.5	0.7	80
Canada	250	NI	NI	NI	100	150	500	20	3	0.8	200
Bulgaria	65	NI	NI	20	46	34	88	10	0.4	0.03	26
UK	130	NI	NI	NI	130	NI	NI	32	10	10	450
Australia	50	NI	NI	NI	60	100	200	20	3	1	300
German	60	NI	NI.	NI	50	40	150	50	1	0.5	70
Poland	100	NI	NI	50	100	100	300	NI	3	NI	100

^a^NI = not indicated.

## Data Availability

The data used to support the findings of this study are included within the supplementary information files.

## Supplementary Materials

Supplementary Materials Tables (S1–S4) represent the Supplementary Information from analytical data that were used to explain the assessment of risk in the studied area. For each sampling area category, the mean concentration (±standard deviation), site ID, and location have been duly given. .

## References

[1] Jerie S., Sibanda E. (2010). The environmental effects of effluent disposal at gold mines in Zimbabwe: a case study of tiger reef mine in kwekwe. *Journal of Sustainable Development in Africa*.

[2] Warren-Hicks W., Parkhurst B. R., Baker S. S. (1989). *Ecological Assessment of Hazardous Waste Sites: A Field and Laboratory Reference*.

[3] Kivyiro D. (2017). *Foreign Direct Investments and Technology Transfer in Tanzania: A Case Study of Geita Gold Mining*.

[4] Banzi F. P., Msaki P. K., Mohammed N. K. (2015). Distribution of heavy metals in soils in the vicinity of the proposed mkuju uranium mine in Tanzania. *Environment and Pollution*.

[5] Lane T. W., Morel F. M. M. (2000). A biological function for cadmium in marine diatoms. *Proceedings of the National Academy of Sciences*.

[6] Rwiza M. J., Kim K.-W., Kim S.-d. (2016). Geochemical distribution of trace elements in groundwater from the north mara large-scale gold mining area of Tanzania. *Groundwater Monitoring & Remediation*.

[7] Mnali S. (2001). Assessment of heavy metal pollution in the lupa gold field, SW Tanzania. *Tanzania Journal of Science*.

[8] Kamunda C., Mathuthu M., Madhuku M. (2016). An assessment of radiological hazards from gold mine tailings in the province of Gauteng in South Africa. *International Journal of Environmental Research and Public Health*.

[9] Chiroma T., Ebewele R., Hymore F. (2014). Comparative assessment of heavy metal levels in soil, vegetables and urban grey waste water used for irrigation in Yola and Kano. *International Refereed Journal of Engineering and Science*.

[10] Means B. (1989). *Risk-Assessment Guidance for Superfund Volume 1. Human Health Evaluation Manual. Part A. Interim Report (Final)*.

[11] Hu H. (2002). *Human Health and Heavy Metals Exposure Life Support: The Environment and Human Health*.

[12] IOMC and UNEP (2002). *Global Mercury Assessment, in Inter-Organization Programme for the Sound Management of Chemicals*.

[13] Podsiki C. (2008). Heavy metals, their salts, and other compounds: a quick reference guide from AIC and the Health & Safety Committee. *AIC News*.

[14] Bose-O’Reilly S., Drasch G., Beinhoff C. (2010). Health assessment of artisanal gold miners in Tanzania. *Science of the Total Environment*.

[15] Ngole-Jeme V. M., Ekosse G.-I. E., Songca S. P. (2018). An analysis of human exposure to trace elements from deliberate soil ingestion and associated health risks. *Journal of Exposure Science & Environmental Epidemiology*.

[16] Khan K., Lu Y., Khan H. (2013). Heavy metals in agricultural soils and crops and their health risks in Swat district, northern Pakistan. *Food and Chemical Toxicology*.

[17] Wang X. (2005). Health risks of heavy metals to the general public in Tianjin, China via consumption of vegetables and fish. *Science of the Total Environment*.

[18] Nyanza E. C., Dewey D., Thomas D. S. K., Davey M., Ngallaba S. E. (2014). Spatial distribution of mercury and arsenic levels in water, soil and cassava plants in a community with long history of gold mining in Tanzania. *Bulletin of Environmental Contamination and Toxicology*.

[19] Cao H., Chen J., Zhang J., Zhang H., Qiao L., Men Y. (2010). Heavy metals in rice and garden vegetables and their potential health risks to inhabitants in the vicinity of an industrial zone in Jiangsu, China. *Journal of Environmental Sciences*.

[20] Arfaenia H. (2016). Assessment of sediment quality based on acid-volatile sulfide and simultaneously extracted metals in heavily industrialized area of Asaluyeh, Persian Gulf: concentrations, spatial distributions, and sediment bioavailability/toxicity. *Environmental Science and Pollution Research*.

[21] Różański S. Ł., Castejón J. M. P., McGahan D. G. (2021). Child risk assessment of selected metal (loid) s from urban soils using in vitro UBM procedure. *Ecological Indicators*.

[22] Kusin F. M., Azani N. N. M., Hasan S., Sulong N. A. (2018). Distribution of heavy metals and metalloid in surface sediments of heavily-mined area for bauxite ore in Pengerang, Malaysia and associated risk assessment. *Catena*.

[23] Masto R. E., George J., Rout T. K., Ram L. C. (2017). Multi element exposure risk from soil and dust in a coal industrial area. *Journal of Geochemical Exploration*.

[24] Organization WHO (2011). *Adverse Health Effects of Heavy Metals in Children*.

[25] Ngole-Jeme V. M., Fantke P. (2017). Ecological and human health risks associated with abandoned gold mine tailings contaminated soil. *Plos One*.

[26] Veronica (2016). An analysis of human exposure to trace elements from deliberate soil ingestion and associated health risks. *Journal of Exposure Science and Environmental Epidemiology*.

[27] Henckel J., Poulsen K. H., Sharp T., Spora P. (2016). Lake victoria goldfields. *Episodes*.

[28] ECA (2008). *Promoting Mineral Clusters: The Case of Tanzania*.

[29] Carter M. R., Gregorich E. G. (2007). *Soil Sampling and Methods of Analysis*.

[30] USEPA (2001). *Risk Assessment Guidance for Superfund: Volume III Part A, Process for Conducting Probabilistic Risk Assessment*.

[31] Bakshi S., Banik C., Banik C., He Z. (2018). The impact of heavy metal contamination on soil health. *Burleigh Dodds Series in Agricultural Science in Managing Soil Health for Sustainable Agriculture*.

[32] Odukoya A. M., Olobaniyi S. B., Oluseyi T. O. (2018). Assessment of potentially toxic elements pollution and human health risk in soil of ilesha gold mining site, southwest Nigeria. *Journal of the Geological Society of India*.

[33] Wuana R. A., Okieimen F. E. (2011). Heavy metals in contaminated soils: a review of sources, chemistry, risks and best available strategies for remediation. *ISRN Ecology*.

[34] Arfaeinia H., Dobaradaran S., Moradi M. (2019). The effect of land use configurations on concentration, spatial distribution, and ecological risk of heavy metals in coastal sediments of northern part along the Persian Gulf. *Science of the Total Environment*.

