# Supplementary material for: Health Risk Assessment of Trace Elements in Soil for People Living and Working in a Mining Area
Source: J Environ Public Health. 2021 Jul 2;2021:9976048. doi: 10.1155/2021/9976048 (PMC8270695; doi:10.1155/2021/9976048)
Supplement: Supplementary Materials — Tables (S1–S4) represent the Supplementary Information from analytical data that were used to explain the assessment of risk in the studied area. For each sampling area category, the mean concentration (±standard deviation), site ID, and location have been duly given. . [file 9976048.f1.docx]

9976048: Health risk assessment of trace elements in soil for people living and working in a mining area

Supplementary Material

The following tables (S1-S4) represent the supplementary material from analytical data that were used to explain the assessment of risk in the studied area.

**Table S1** Levels of trace elements in soil samples collected from small-scale mining pits

| Site ID | Location coordinate | Mean elemental concentration (±SD) (mg/kg) | | | | | | | | |
| --- | --- | --- | --- | --- | --- | --- | --- | --- | --- | --- |
|  |  | Cr | Co | Ni | Cu | Zn | As | Cd | Hg | Pb |
| MD1 | S03⁰06.067’ E032⁰04.016′ | 60.31±2.99 | 19.24±2.73 | 94.74±3.61 | 196.43±3.24 | 148.82±3.72 | 6.77±2.78 | 6.05±2.78 | 0 | 6.67±2.31 |
| MD2 | S03⁰06.075’ E032⁰04.000’ | 59.69±1.29 | 12.48±4.55 | 100.89±3.81 | 134.84±1.10 | 146.91±1.78 | 10.74±3.32 | 2.01±0.21 | 0.07 | 18.32±1.53 |
| MD3 | S03⁰06.071’ E032⁰03.988’ | 46.12±6.68 | 24.60±1.68 | 118.31±4.82 | 182.46±4.20 | 148.87±1.10 | 13.46±3.02 | 10.30±0.02 | 0 | 9.68±1.40 |
| MD4 | S03⁰06.076′ E032⁰03.983′ | 41.76±5.13 | 18.55±3.19 | 86.37±4.85 | 237.46±3.30 | 151.31±1.04 | 10.89±2.09 | 5.21±2.02 | 0 | 4.19±0.46 |
| MD5 | S03⁰06.070′ E032⁰03.969’ | 113.23±4.34 | 14.07±3.82 | 78.32±2.91 | 150.10±3.10 | 87.78±1.81 | 14.63±3.45 | 0.03±0.01 | 0 | 15.08±0.54 |
| MD6 | S03⁰06.069’ E032⁰03.959’ | 53.58±2.03 | 14.17±2.42 | 95.36±1.25 | 214.26±0.87 | 133.43±3.48 | 10.74±2.89 | 3.13±2.43 | 0.09 | 5.40±0.97 |
| MD7 | S03⁰06.062’ E032⁰03.952’ | 86.86±3.41 | 11.66±2.27 | 84.33±4.97 | 118.37±1.15 | 108.83±0.76 | 14.03±3.08 | 4.78±1.23 | 0 | 7.95±1.21 |
| MD8 | S03⁰06.062’ E032⁰03.867’ | 53.87±10.38 | 27.21±2.76 | 89.03±4.39 | 737.66±1.30 | 150.16±2.63 | 0 | 7.14±2.95 | 0 | 15.74±0.86 |
| MD9 | S03⁰06.070’ E032⁰03.949’ | 55.13±7.56 | 13.83±3.49 | 97.88±3.23 | 152.64±6.47 | 133.78±1.66 | 8.73±1.98 | 7.86±3.10 | 0 | 4.58±0.49 |
| MD10 | S03⁰06.081’ E032⁰03.934’ | 51.85±0.75 | 20.37±1.28 | 128.40±4.06 | 178.36±5.39 | 154.25±5.59 | 25.9±4.89 | 10.45±0.77 | 0 | 7.66±0.59 |
| MD11 | S03⁰06.081’ E032⁰03.934’ | 45.29±4.17 | 18.90±5.11 | 89.97±9.00 | 172.07±2.41 | 165.46±1.91 | 5.48±0.08 | 6.32±00 | 0 | 3.04±1.11 |
| MD12 | S03⁰06.080’ E032⁰03.921’ | 63.31±4.76 | 10.63±2.27 | 67.88±3.22 | 133.27±2.81 | 126.98±0.94 | 17.73±4.87 | 6.09±2.21 | 0 | 4.58±0.74 |
| MD13 | S03⁰06.085’ E032⁰03.913’ | 46.91±2.74 | 10.68±0.86 | 44.65±4.77 | 51.86±2.86 | 126.04±1.89 | 5.94±0.96 | 5.32±1.23 | 0 | 4.09±0.13 |
| MD14 | S03⁰06.090’ E032⁰03.913’ | 40.23±1.67 | 12.10±2.57 | 87.13±2.27 | 167.87±4.26 | 126.98±1.68 | 18.79±5.12 | 7.60±0.01 | 0 | 4.35±0.15 |
| MD15 | S03⁰06.098’ E032⁰03.902’ | 75.35±0.23 | 19.63±2.82 | 97.98±6.71 | 155.85±3.05 | 108.65±0.13 | 8.09±3.36 | 9.21±0.81 | 0 | 3.34±0.61 |
| MD16 | S03⁰06.187’ E032⁰03.716’ | 41.95±6.14 | 22.59±2.60 | 138.39±3.84 | 170.06±4.68 | 149.49±3.67 | 22.8±4.32 | 9.01±0.00 | 0 | 4.32±0.61 |
| MD17 | S03⁰06.185’ E032⁰03.707’ | 34.45±2.09 | 20.72±5.61 | 88.55±1.67 | 184.34±1.94 | 157.28±2.00 | 3.89±0.09 | 13.98±2.42 | 0 | 4.32±2.25 |
| MD18 | S03⁰06.184’ E032⁰03.706’ | 52.32±4.66 | 24.21±5.99 | 98.43±2.59 | 149.19±1.52 | 172.14±1.74 | 6.99±1.43 | 6.36±2.07 | 0 | 7.33±0.72 |
| MD19 | S03⁰06.188’ E032⁰03.698’ | 47.31±0.62 | 19.14±1.52 | 122.25±6.00 | 108.09±3.07 | 142.68±4.03 | 9.53±1.23 | 7.31±2.10 | 0.06 | 3.14±0.87 |
| MD20 | S03⁰06.184’ E032⁰03.695’ | 41.63±5.98 | 17.57±1.56 | 108.46±4.61 | 167.97±3.44 | 215.43±2.73 | 4.12±0.05 | 10.69±1.29 | 0.006 | 1.77±0.17 |
| MD21 | S03⁰06.188’ E032⁰03.693’ | 46.07±7.35 | 21.65±0.73 | 111.32±1.92 | 158.75±2.14 | 125.38±4.47 | 16.34±3.65 | 10.59±0.55 | 0 | 6.12±1.08 |
| MD22 | S03⁰06.214’ E032⁰03.660’ | 48.46±4.59 | 11.81±2.47 | 70.61±3.97 | 139.04±7.58 | 155.45±0.41 | 8.09±2.61 | 9.83±2.12 | 0 | 1.60±0.34 |
| MD23 | S03⁰06.221’ E032⁰03.653’ | 70.48±9.07 | 18.60±4.35 | 100.02±2.93 | 126.54±4.01 | 134.36±2.61 | 4.73±0.13 | 9.59±2.57 | 3.72 | 3.96±1.99 |
| MD24 | S03⁰06.218’ E032⁰03.651’ | 61.55±3.29 | 15.40±2.79 | 125.15±1.89 | 166.81±6.47 | 131.96±2.54 | 6.28±1.43 | 8.78±2.65 | 0 | 3.24±1.20 |
| MD25 | S03⁰06.219’ E032⁰03.659’ | 47.23±0.92 | 26.92±0.52 | 91.73±2.12 | 179.60±5.55 | 116.52±1.67 | 11.68±2.85 | 14.09±1.64 | 0 | 6.41±1.00 |
| MD26 | S03⁰06.220’ E032⁰03.673’ | 39.39±7.30 | 21.99±2.80 | 99.40±1.71 | 130.91±0.82 | 130.18±0.60 | 36.11±5.98 | 7.48±2.10 | 0 | 2.45±0.35 |
| MD27 | S03⁰06.216’ E032⁰03.672’ | 37.32±10.92 | 11.75±3.26 | 87.30±8.98 | 145.26±2.27 | 117.06±1.89 | 20.76±3.43 | 6.98±1.07 | 0 | 4.55±0.34 |
| MD28 | S03⁰06.210’ E032⁰03.680’ | 52.61±3.45 | 19.24±1.26 | 73.44±1.66 | 153.28±4.94 | 58.02±.04 | 11.68±3.19 | 7.98±2.16 | 0 | 8.64±0.64 |
| MD29 | S03⁰06.217’ E032⁰03.686’ | 56.16±7.70 | 14.91±1.15 | 131.61±2.75 | 173.34±1.39 | 141.79±2.03 | 34.49±5.86 | 7.98±2.20 | 0 | 2.29±0.72 |
| Minimum | | 34.45±2.09 | 10.63±2.27 | 44.65±4.77 | 51.86±2.86 | 58.02±.04 | 0 | 6.03±0.01 | 0 | 1.60±0.34 |
| Maximum | | 113.23±4.34 | 26.92±0.52 | 131.61±2.75 | 737.66±1.30 | 215.43±2.73 | 36.11±0.47 | 9.83±2.12 | 3.72 | 18.32±1.53 |
| Overall mean for all sampling sites | | 54.15±4.56 | 17.75±2.73 | 96.82±3.81 | 177.16±3.29 | 136.76±2.22 | 12.73827586 | 7.86±1.03 | 0.07 | 6.03±0.91 |

**Table S2** Levels of trace elements in soil samples collected from a small-scale mine site at an area “A” dominated by mineral washing activities

| Site ID | Location coordinate | Mean elemental concentration (±SD) (mg/kg) | | | | | | | | |
| --- | --- | --- | --- | --- | --- | --- | --- | --- | --- | --- |
|  |  | Cr | Co | Ni | Cu | Zn | As | Cd | Hg | Pb |
| WA1 | S03⁰06.181’ E032⁰03.904’ | 73.48±2.01 | 20.32±2.29 | 70.61±3.95 | 146.18±1.11 | 104.55±1.14 | 14.71±0.76 | 0 | 0 | 11.39±0.90 |
| WA2 | S03⁰06.199’ E032⁰03.894’ | 66.79±5.48 | 24.60±7.71 | 97.53±2.61 | 137.09±0.93 | 137.26±4.19 | 9.42±1.45 | 8.77±1.05 | 0 | 11.29±0.36 |
| WA3 | S03⁰06.189’ E032⁰03.869’ | 60.63±3.83 | 21.5±3.54 | 90.48±1.04 | 127.15±2.20 | 130.54±4.28 | 9.91±0.33 | 7.58±1.43 | 0 | 9.42±0.19 |
| WA4 | S03⁰06.162’ E032⁰03.854’ | 120.19±8.98 | 15.35±3.54 | 101.68±3.31 | 178.97±2.46 | 145.13±1.59 | 7.26±0.11 | 0 | 0 | 3.04±0.20 |
| WA5 | S03⁰06.178’ E032⁰03.836’ | 90.48±3.34 | 15.65±0.90 | 81.64±0.81 | 129.88±1.65 | 98.28±1.67 | 20.46±0.77 | 0 | 0 | 10.27±0.82 |
| WA6 | S03⁰06.173’ E032⁰03.845’ | 96.07±7.26 | 12.63±7.74 | 47.51±2.14 | 74.17±2.58 | 50.49±1.60 | 6.49±2.20 | 5.78±0.75 | 0 | 17.3±2.30 |
| WA7 | S03⁰06.203’ E032⁰03.848’ | 49.39±4.91 | 14.76±1.60 | 98.23±6.30 | 130.98±1.71 | 145.75±6.84 | 15.16±0.69 | 0 | 0 | 3.27±0.25 |
| WA8 | S03⁰06.027’ E032⁰03.870’ | 56.48±7.80 | 12.20±0.89 | 98.16±4.88 | 131.22±2.78 | 142.37±0.60 | 8.02±0.43 | 0 | 0 | 2.45±0.20 |
| WA9 | S03⁰06.049’ E032⁰03.876’ | 72.01±7.45 | 18.11±1.85 | 97.91±1.57 | 162.85±3.55 | 128.62±2.14 | 24.09±1.35 | 0 | 0 | 10.89±0.98 |
| WA10 | S03⁰06.641’ E032⁰03.861’ | 59.29±5.70 | 31.89±12.32 | 75.86±1.87 | 118.71±0.77 | 115.32±4.40 | 7.83±1.31 | 4.75±0.73 | 0 | 6.97±1.86 |
| WA11 | S03⁰06.031’ E032⁰03.862’ | 109.08±1.90 | 28.69±1.63 | 80.11±2.50 | 183.00±7.08 | 105.53±2.13 | 21.74±1.71 | 0 | 0 | 24.44±1.63 |
| WA12 | S03⁰06.030’ E032⁰03.844’ | 102.12±7.54 | 20.62±2.82 | 46.49±1.50 | 108.26±3.94 | 39.78±0.74 | 10.78±1.94 | 0 | 0 | 17.54±1.31 |
| WA13 | S03⁰06.019’ E032⁰03.835’ | 67.19±6.17 | 24.9±1.40 | 102.34±2.55 | 191.24±2.83 | 125.42±4.47 | 2.67±0.08 | 7.3±0.73 | 0.05 | 10.67±1.21 |
| WA14 | S03⁰06.041’ E032⁰03.812’ | 93.02±7.14 | 37.3±15.96 | 77.04±6.70 | 138.12±3.64 | 112.38±3.60 | 8.28±0.49 | 4.21±1.93 | 0 | 9.95±0.97 |
| WA15 | S03⁰06.057’ E032⁰03.804’ | 69.64±14.88 | 38.73±12.19 | 82.57±9.42 | 154.79±4.12 | 122.31±2.90 | 10.1±0.52 | 5.83±1.55 | 0 | 9.03±0.17 |
| WA16 | S03⁰06.097’ E032⁰03.764’ | 67.72±4.02 | 10.09±0.90 | 80.15±1.70 | 153.05±3.60 | 139.7±1.56 | 21.63±0.46 | 3.1±0.00 | 0 | 5.17±0.11 |
| WA17 | S03⁰06.994’ E032⁰03.879’ | 55.99±1.87 | 12.7±1.54 | 95.94±3.73 | 128.21±3.16 | 138.19±2.19 | 10.74±1.48 | 0 | 0.053 | 4.45±0.30 |
| WA18 | S03⁰06.967’ E032⁰03.845’ | 86.99±4.26 | 23.03±1.64 | 80.36±1.30 | 144.06±2.52 | 96.68±1.84 | 14.67±0.33 | 5.85±1.63 | 0 | 11.55±1.33 |
| Minimum | | 49.39±4.91 | 10.09±0.90 | 46.49±1.50 | 74.17±2.58 | 39.78±0.74 | 6.49±2.20 | 0 |  | 2.45±0.20 |
| Maximum | | 120.19±8.98 | 38.73±12.19 | 101.68±3.31 | 178.97±2.46 | 145.75±6.84 | 24.09±1.35 | 8.77±1.05 |  | 17.54±1.31 |
| Overall mean for all sampling sites | | 77.59±5.81 | 21.284.14 | 83.59±3.21 | 140.99±2.81 | 115.46±2.66 | 12.44±0.91 | 6.34±1.16 | 0.05 | 9.95±0.84 |

**Table S3** Levels of trace elements in soil samples collected from a small-scale mine site at an area “B” dominated by mineral washing activities

| Site ID | Location coordinate | Mean elemental concentration (±SD) (mg/kg) | | | | | | | | |
| --- | --- | --- | --- | --- | --- | --- | --- | --- | --- | --- |
|  |  | Cr | Co | Ni | Cr | Zn | As | Cr | Hg | Pb |
| WB1 | S03⁰06.178’ E032⁰03.904’ | 102.66±2.85 | 32.43±3.92 | 56.16±2.13 | 176.65±4.21 | 66.91±2.68 | 14.71±2.17 | 3.63±0.31 | 3.67 | 31.11±1.41 |
| WB2 | S03⁰06.198’ E032⁰03.893’ | 69.87±0.70 | 21.95±9.61 | 52.26±2.69 | 98.04±4.62 | 73.77±6.23 | 8.17±1.29 | 6.11±2.34 | 3.59 | 10.63±1.41 |
| WB3 | S03⁰06.188’ E032⁰03.858’ | 47.15±2.79 | 10.14±1.64 | 51.74±1.71 | 74.71±3.86 | 72.57±1.54 | 15.31±0.39 | 0 | 3.59 | 4.55±0.60 |
| WB4 | S03⁰06.162’ E032⁰03.854’ | 52.73±3.73 | 28.54±3.66 | 53.29±4.85 | 85.78±6.85 | 70.03±3.97 | 8.28±1.20 | 7.25±1.36 | 3.71 | 9.23±1.23 |
| WB5 | S03⁰06.178’ E032⁰03.836’ | 210.4±10.39 | 35.33±2.22 | 99.06±2.69 | 157.08±4.80 | 69.85±2.30 | 15.88±0.30 | 5.56±1.84 | 3.58 | 23.13±0.48 |
| WB6 | S03⁰06.173’ E032⁰03.845’ | 280±12.45 | 15.5±0.97 | 69.82±3.20 | 230.66±3.99 | 88.89±1.62 | 44.13±0.79 | 2.26±0.00 | 3.62 | 10.67±0.48 |
| WB7 | S03⁰06.204’ E032⁰03.847’ | 55.1±7.68 | 18.69±3.28 | 105.59±0.79 | 130.12±1.92 | 135.69±2.74 | 21.21±1.31 | 8.48±1.63 | 3.43 | 7.26±0.34 |
| WB8 | S03⁰06.029’ E032⁰03.872’ | 93.96±5.31 | 26.08±4.26 | 62.52±2.49 | 146.32±3.88 | 84.58±1.94 | 21.4±1.21 | 6.83±2.37 | 3.55 | 26.04±0.82 |
| WB9 | S03⁰06.049’ E032⁰03.873’ | 70.13±1.29 | 14.71±1.68 | 75.28±2.37 | 109.69±3.45 | 131.92±1.36 | 19.44±0.65 | 0 | 3.72 | 4.15±1.45 |
| WB10 | S03⁰06.641’ E032⁰03.861’ | 114.03±14.47 | 18.31±8.13 | 70.23±3.30 | 155.61±3.55 | 85.65±0.39 | 60.54±1.16 | 0 | 3.61 | 14.36±0.91 |
| WB11 | S03⁰06.031’ E032⁰03.862’ | 84.86±7.36 | 36.76±5.72 | 60.86±2.85 | 170.09±3.32 | 68.16±1.78 | 24.65±0.88 | 3.27±0.70 | 3.08 | 34.19±2.52 |
| WB12 | S03⁰06.030’ E032⁰03.844’ | 66.39±1.62 | 10.73±0.81 | 69.23±3.80 | 118.75±1.71 | 94.05±1.82 | 16.22±0.89 | 0 | 3.68 | 9.65±0.50 |
| WB13 | S03⁰06.018’ E032⁰03.836’ | 93.29±4.02 | 23.37±1.13 | 78.25±3.18 | 143.21±2.56 | 119.15±3.55 | 11.87±1.35 | 7.38±1.17 | 3.66 | 8.54±1.06 |
| WB14 | S03⁰06.041’ E032⁰03.813’ | 87.27±4.57 | 21.01±2.00 | 92.63±4.92 | 159.95±0.85 | 142.82±1.14 | 25.07±1.64 | 0 | 3.28 | 10.31±0.85 |
| WB15 | S03⁰06.056’ E032⁰03.804’ | 45.73±2.86 | 9.2±0.76 | 51.95±1.40 | 92.1±6.49 | 80.66±1.29 | 11.3±0.62 | 2.26±0.00 | 3.65 | 5.33±0.44 |
| WB16 | S03⁰06.097’ E032⁰03.764’ | 61.43±1.75 | 17.52±2.15 | 96.08±3.86 | 155.33±2.88 | 157.85±3.63 | 11.91±0.49 | 2.08±0.00 | 3.7 | 5.99±0.10 |
| WB17 | S03⁰06.991’ E032⁰03.880’ | 66.65±3.29 | 11.33±3.12 | 82.39±1.20 | 131.35±2.26 | 136.72±0.86 | 15.13±0.52 | 0 | 0.81 | 2.78±0.48 |
| Minimum | | 45.73±2.86 | 9.2±0.76 | 51.74±1.71 | 74.71±3.86 | 66.91±2.68 | 8.17±1.29 | 0 | 0.81 | 2.78±0.48 |
| Maximum | | 280±12.45 | 36.76±5.72 | 105.59±0.79 | 230.66±3.99 | 157.85±3.63 | 60.54±1.16 | 8.48±1.63 | 3.72 | 34.19±2.52 |
| Overall mean for all sampling sites | | 94.23±5.13 | 20.68±3.24 | 72.19±2.79 | 137.38±3.60 | 98.78±2.29 | 20.31±0.99 | 6.98±0.34 | 3.41±0.69 | 12.82±0.89 |

**Table S4** Levels of trace elements in soil samples collected from a control area, which had minimal mining activities

| Site ID | Location coordinate | Mean elemental concentration (±SD) (mg/kg) | | | | | | | | |
| --- | --- | --- | --- | --- | --- | --- | --- | --- | --- | --- |
|  |  | Cr | Co | Ni | Cr | Zn | As | Cr | Hg | Pb |
| C1 | S03⁰06.490’ E032⁰03.160’ | 49.49±3.32 | 8.87±1.09 | 50.80±3.34 | 60.84±1.72 | 34.48±1.74 | 0.5 | 9.27±1.13 | 0 | 14.10±3.22 |
| C2 | S03⁰06.280’ E032⁰03.336’ | 53.01±3.56 | 30.21±2.63 | 85.78±6.09 | 141.16±3.84 | 160.75±3.19 | 0 | 6.86±1.84 | 0 | 12.79±2.32 |
| C3 | S03⁰06.225’ E032⁰03.372’ | 40.08±2.89 | 11.51±2.32 | 123.15±0.33 | 101.46±10.77 | 44.45±3.03 | 0.51 | 6.68±0.06 | 0 | 11.45±0.74 |
| C4 | S03⁰06.223’ E032⁰03.378’ | 56.57±4.72 | 24.80±4.09 | 79.70±7.60 | 113.96±9.87 | 82.79±3.32 | 0.514±0.056 | 7.04±1.90 | 0 | 12.40±2.32 |
| C5 | S03⁰06.229’ E032⁰03.370’ | 40.09±2.26 | 16.2±1.04 | 84.54±6.35 | 127.43±9.05 | 80.42±6.31 | 0.48 | 7.81±1.30 | 0 | 13.50±2.02 |
| C6 | S03⁰06.272’ E032⁰03.329’ | 49.6±2.34 | 15.9±2.80 | 92.98±3.12 | 122.3±7.21 | 83.86±4.78 | 0.49 | 8.17±0.90 | 0 | 16.00±1.98 |
| C7 | S03⁰06.298’ E032⁰03.360’ | 49.89±2.0 | 11.23±2.13 | 119.09±9.14 | 90.45±5.01 | 98.53±7.12 | 0.5 | 8.02±1.89 | 0 | 16.46±0.65 |
| C8 | S03⁰06.190’ E032⁰03.218’ | 51.98±3.98 | 27.8±4.21 | 50.81±2.52 | 87.47±5.09 | 36.52±12.10 | 0.41 | 7.03±0.81 | 0 | 16.10±0.98 |
| C9 | S03⁰03.218’ E032⁰03.207’ | 56.58±4.62 | 22.9±3.31 | 79.89±5.21 | 89.86±4.12 | 82.9±5.25 | 0.49±0.065 | 6.69±0.87 | 0 | 12.2±0.87 |
| C10 | S03⁰06.350’ E032⁰03.290’ | 50.87±3.08 | 19.09±1.98 | 81.89±7.68 | 108.43±6.32 | 111.54±8.62 | 0.47 | 7.13±0.11 | 0 | 12.00±1.32 |
| Minimum | | 40.09±2.26 | 8.87±1.09 | 50.81±2.52 | 60.84±1.72 | 34.48±1.74 | 0 | 6.86±1.84 | 0 | 11.45±0.74 |
| Maximum | | 56.58±4.62 | 30.21±2.63 | 123.15±0.33 | 141.16±3.84 | 160.75±3.19 | 0.514±0.056 | 9.27±1.13 | 0 | 16.46±0.65 |
| Overall mean for all sampling sites | | 49.79±5.81 | 18.85±2.76 | 84.86±3.21 | 104.36±2.81 | 80.62±2.66 | 0.51±0.06 | 7.47±0.96 | 0 | 13.70±0.84 |
